# Evaluation of Low-Molecular-Weight Heparin for Treatment of Portal Vein Thrombosis in Liver Cirrhosis Patients

**DOI:** 10.3390/medicina59020292

**Published:** 2023-02-04

**Authors:** Ji Min Han, Youngil Koh, Sung Hwan Kim, Sung Yun Suh, Yoon Sook Cho, Jeong-Hoon Lee, Su Jong Yu, Jung-Hwan Yoon, Hye Sun Gwak

**Affiliations:** 1 College of Pharmacy, Chungbuk National University, Cheongju 28160, Republic of Korea; 2 Department of Pharmacy, Seoul National University Hospital, Seoul 03080, Republic of Korea; 3 Department of Internal Medicine, College of Medicine, Seoul National University, Seoul 03080, Republic of Korea; 4 Cancer Research Institute, College of Medicine, Seoul National University, Seoul 03080, Republic of Korea; 5 Liver Research Institute, College of Medicine, Seoul National University, Seoul 03080, Republic of Korea; 6 College of Pharmacy and Graduate School of Pharmaceutical Sciences, Ewha Womans University, Seoul 03760, Republic of Korea

**Keywords:** portal vein thrombosis, dalteparin, low-molecular-weight heparin, liver cirrhosis

## Abstract

*Background and Objectives:* Even though low-molecular-weight heparin (LMWH), including dalteparin, has a critical role in portal vein thrombosis (PVT) treatment in liver cirrhosis (LC) patients, the predictive factors and the proper dose of dalteparin for PVT treatment and relapse have not yet been investigated. *Materials and Methods:* This retrospective study evaluated the records of LC patients receiving dalteparin from July 2013 to June 2019. The odds ratio (OR) and adjusted OR were calculated from univariate and multivariable analyses, respectively. *Results:* Among data from 121 patients, the overall recanalization rate of all patients was 66.1% (80 patients). No history of variceal bleeding (OR 4.6, 95% CI: 1.88–11.43) and the case of newly developed thrombus before dalteparin treatment (OR 3.2, 95% CI: 1.24–8.08) were predictive factors associated with increased treatment response. Relapse of PVT occurred in 32 out of 80 patients (40%) who showed a recanalization. The risk of relapse was 3.1–3.9 times higher in those who took more than three months or more than six months from the diagnosis of PVT to dalteparin treatment compared to those who took less than these durations, respectively. In the dosing regimen, patients with the kg-based dosing regimen showed 2.6 times better response than those with the fixed dosing regimen. However, no difference in bleeding complications was observed. *Conclusion:* In the dosing regimen, the kg-based regimen that was the same as the venous thromboembolism regimen was a better option for the efficacy and safety of dalteparin therapy. Additionally, when treating PVT in LC patients, careful monitoring is recommended for patients with predictive factors for treatment response and relapse of PVT.

## 1. Introduction

Portal vein thrombosis (PVT) in patients with liver cirrhosis (LC) has a high incidence of 10–26% [1,2]. PVT can lead to fatal symptoms such as abdominal pain, variceal bleeding, and intestinal infarction, causing death in severe cases. Considering that the mortality rate of patients with LC or hepatocellular carcinoma increases by 26% compared to that of PVT itself (10%), the treatment of PVT has a critical role in patients with LC [3]. Although there is a tendency for coagulation to commonly occur in LC patients, a decrease in the synthesis of substances involved in the coagulation cascades, such as protein C and protein S, has also been reported [4,5]. Thus, this indicates that cirrhosis patients are more likely to develop blood clots and bleeding. In the LC state, both bleeding and coagulation tendencies can occur, so caution is needed regarding bleeding during the treatment of PVT [3].

According to the European Association for the Study of the Liver (EASL) guideline, anticoagulation therapies, including low-molecular-weight heparin (LMWH), are recommended for the treatment of PVT in LC patients [6]. Several research studies demonstrated that anticoagulation therapy effectively recanalizes portal veins and reduces thrombus progression risk [7,8,9,10]. Although LC patients usually have risk factors for increased bleeding tendency, such as coagulopathy of liver disease and thrombocytopenia, the therapeutic dose of anticoagulation did not show a significant increase in bleeding risk among LC patients with PVT [9].

Among anticoagulant medications, vitamin K antagonist (VKA) has been used effectively for a long period. However, there are several concerns about using VKA for LC patients with PVT. VKA is a medication that requires frequent dose adjustments due to its narrow therapeutic range [11]. In addition, the unique condition of simultaneously promoting coagulation and bleeding in LC patients makes it difficult to predict the risk of bleeding with the international normalized ratio (INR) and prothrombin time [8]. Accordingly, LMWH has been mainly prescribed for PVT treatment of LC patients. 

Since there is currently no established therapeutic dose of LMWH for PVT, it is administered based on the dose of venous thromboembolism (VTE). However, in clinical practice, the dose is frequently reduced due to the coagulopathy-related bleeding risk of LC patients or the occurrence of bleeding side effects during treatment. In addition, the dose change caused by the difference between the loading dose and the maintenance dose increases the possibility of medication error [7]. Although it is necessary to evaluate the efficacy of LMWH for PVT according to the dose, research has rarely been conducted. 

This study aims to analyze the predictive factors for PVT treatment and relapse using dalteparin and establish the appropriate dose of dalteparin.

## 2. Methods and Materials

### 2.1. Patients

This retrospective study was performed using medical records from July 2013 to June 2019 at Seoul National University Hospital, Korea. Eligible patients were older than 18 years and LC patients with secondary bland PVT who received dalteparin for anticoagulation treatment. Diseases that cause LC include alcoholic liver disease, hepatitis B or C, and primary biliary cirrhosis, among others. Three patients had metastatic hepatocellular carcinoma (HCC). Patients with hepatic cirrhosis due to venous obstruction or thrombosis-related disorders, such as Budd–Chiari syndrome, were excluded. PVT was assessed by computed tomography (CT) or magnetic resonance imaging (MRI). 

For response evaluations, follow-up imaging tests were conducted using CT or MRI every three months or when clinically relevant events occurred. The treatment responses were divided into the following groups: (1) complete recanalization was defined by the complete disappearance of the intravenous thrombus, (2) partial recanalization was defined by the reduced but remaining thrombus at >25% based on the cross-section of the vessel, (3) stable disease was defined by the no change or decrease in the thrombus volume of <25% of the cross-section of the vessel, (4) progressive status was defined by the increased thrombus size. The overall recanalization rate was determined as the sum of the fraction of patients who had complete or partial recanalization. Furthermore, ‘poor recanalization’ included both stable and progressive diseases. 

Patients who showed complete or partial recanalization were included in the response group after receiving dalteparin. The relapse was defined as the occurrence of PVT again after six-month therapy of dalteparin in the response group. 

### 2.2. Anticoagulation Protocol

Patients received dalteparin according to two protocols: the kg-based and fixed dosing regimens. The kg-based dosing regimen was the same as the VTE therapeutic dosing regimen of dalteparin: 200 units/kg once daily during the first four weeks, with a subsequent reduction to 150 units/kg per day. The fixed dosing regimen is a protocol of continuously administering 150 units/kg per day without a loading dose in the VTE therapeutic dosing regimen. The total initial treatment duration was six months. Additionally, the dose and schedule of dalteparin can be changed depending on the patient’s general condition and clinical status. If thrombosis remained or recurred, the dalteparin treatment could be extended or resumed. 

Hemorrhagic complications following the dalteparin regimen were evaluated during the study. The severity of bleeding was determined using the World Health Organization (WHO) bleeding scale, composed of four grades according to symptoms and the needs for red blood cell transfusion within 24 h because of bleeding. In this study, bleeding complications were defined as grade II or higher. 

### 2.3. Statistical Analysis

The chi-squared or Fisher’s exact test was used to compare categorical variables between patients with and without treatment response and relapse. It was also performed to compare categorical variables between two different dosing regimens. Multivariable logistic regression analysis was performed to identify independent predictive factors for treatment response and relapse of PVT. Factors having a *p*-value < 0.1 from the univariate analysis were included in the multivariable analysis. Furthermore, the odds ratio (OR) and adjusted OR were calculated from univariate and multivariate analyses, respectively. 

A *p*-value < 0.05 was considered statistically significant. All statistical analyses were performed with the Statistical Package for the Social Sciences, version 20.0 for Windows (IBM Corp., Armonk, NY, USA). 

## 3. Results

A total of 136 patients treated from July 2013 to June 2019 were eligible for participation in this study. Patients with inappropriate follow-up data after dalteparin administration (*n* = 15) were excluded from this study. Accordingly, data from 121 patients treated with dalteparin were used for the analysis. The median duration of treatment with dalteparin was 177 days (range: 21–387 days).

As shown in Table 1, almost half of the study patients (50.4%) were older than 65 years (age range, 37–85 years), and 93 patients (76.9%) were male. The most common cause of LC was hepatitis B or C, accounting for almost 80%. About 70% of patients had HCC, and 71.1% of patients had experience with topical trans-catheter arterial chemoembolization (TACE) or ratio-frequency ablation (RFA). No patients were administered systemic chemotherapy. Additionally, 80.2% of patients had esophageal or gastric varices, and about half had a history of variceal bleeding. Patients had experienced variceal bleeding for at least one year. When classified according to the Child–Pugh classification, more than half of the patients were class A, and only six were classified as class C. 

The overall recanalization rate for all patients was 66.1% (80 patients). In the univariate analysis, the history of varix bleeding, the state of thrombus before dalteparin administration, the dosing regimen, and the period from the diagnosis of PVT to the start of dalteparin therapy were significant factors for dalteparin treatment response. In the case of laboratory values, the platelet count and prothrombin time before initiation of dalteparin had no significant effect on the response to dalteparin. 

Multivariate analysis was performed by combining the predictive factors with *p*-value <0.1 in univariate analysis and the general factors of sex and age. As shown in Table 2, predictors affecting the response to dalteparin treatment were the history of variceal bleeding and the status of the thrombus at treatment initiation. Patients with no history of variceal bleeding had a 4.6-fold increased treatment response compared to those with a history of variceal bleeding. In addition, in the case of newly developed thrombus before dalteparin treatment, the treatment response was 3.2 times higher than in the progressive thrombus. Patients with the kg-based dosing regimen showed 2.6 times better response than those with the fixed dosing regimen. 

Predictive factors affecting the relapse of PVT were analyzed in patients who had a treatment response to dalteparin. As shown in Table 3, relapse of PVT occurred in 32 out of 80 patients (40%) who showed a recanalization. Notably, a significant factor for PVT relapse was the duration from PVT diagnosis to dalteparin treatment. However, the dosing regimen was not a statistically significant factor in the risk of relapse. In the case of laboratory values, the platelet count and prothrombin time before initiation of dalteparin were not significant factors in PVT relapse.

The multivariable analysis controlled for variables with *p*-values < 0.1 from the univariate analysis and the general factors (sex and age) were performed (Table 4). 

For the analysis of relapse risk, two models were used according to the period from PVT diagnosis to dalteparin treatment. Model I included a three-month duration, in addition to age and sex, and model II included a six-month duration instead of the three-month duration. Notably, the time from PVT diagnosis to dalteparin treatment was a significant factor for increased risk of relapse in both models: three-month period (OR 3.05, 95% CI 1.19–7.83), six-month period (OR 3.89, 95% CI 1.39–10.91).

Table 5 shows the clinical characteristics of two different dosing regimens. After six months of therapy, 25 patients with a fixed dosing regimen and 55 patients with a kg-based regimen reached complete/partial recanalization of PVT. Among clinical characteristics, there were no statistically significant factors related to the choice of dosing regimen except PVT treatment response. Bleeding complications were reported in 14 patients (11.6%): 3 with a fixed dosing regimen and 11 with a kg-based regimen. In bleeding events, there was no significant difference between the dosing regimens. In our study, fatal bleeding due to variceal bleeding occurred in one patient with the fixed dosing regimen. Hematemesis occurred in three patients, including two in the kg-based dosing group and one in the fixed dosing group. Hematochezia occurred in one patient in each dosing group. The remaining eight patients in the kg-based dosing group showed the following: Four patients suffered from melena, and two patients had hematuria. One patient had bleeding from the invasive site, and the last patient had a hematoma at the injection site.

## 4. Discussion

In this study, factors affecting response to PVT treatment and PVT relapse have been investigated, including the choice of the dosing regimen. In the case of a predictive factor for treatment response, patients with no history of variceal bleeding had a 4.6-fold increased treatment response compared to those with a history of variceal bleeding. In addition, the newly developed thrombus showed a 3.2-fold increased treatment response compared to the progressive thrombus. Notably, patients with the kg-based dosing regimen showed 2.6 times better response than those with the fixed dosing regimen. Furthermore, in the case of a predictive factor for PVT relapse, the longer duration from the time of PVT diagnosis to the start of dalteparin treatment (longer than six months or longer than three months) was a significant factor for PVT relapse. 

The efficacy of anticoagulation therapy in PVT has been evaluated through several investigations [7,8,9]. LMWH, VKA, and direct oral anticoagulant (DOAC) are commonly used anticoagulants. VKA requires detecting the INR to predict the anticoagulant effect. Still, the correlation between the INR and the degree of anticoagulation in LC patients has not been established [10,12]. As DOACs become a standard treatment for general patients with thrombosis, recent guidelines mention using DOACs in PVT patients with or without cirrhosis, but the evidence is still limited [13]. Therefore, LMWH, including dalteparin, is still the primary therapy for PVT in LC patients.

A history of variceal bleeding and status of thrombus were significant factors for predicting treatment response in dalteparin therapy. The development of varices is caused by an increase in portal pressure. Gastroesophageal varices can be detected at the time of diagnosis in about 50% of LC patients [14,15,16]. PVT is also associated with portal hypertension, which usually causes varices, and bleeding due to its rupture may also occur. Hence, variceal bleeding is one of the most common symptoms of PVT [17]. Accordingly, a history of variceal bleeding may indicate that it is not the acute phase of PVT. A prospective study on the effect of anticoagulation therapy revealed that chronic PVT (median >200 days after onset) was significantly associated with recanalization failure of the portal vein [18]. Thus, these findings explain why patients without a history of variceal bleeding and those with newly developed PVT showed better treatment responses to dalteparin administration. 

Duration from diagnosis of PVT to dalteparin treatment significantly increased the risk of relapse. Our previous studies using LMWH, including enoxaparin and dalteparin, revealed that the period from PVT diagnosis to starting anticoagulation treatment affected initial treatment outcomes [19]. Another anticoagulation study using LMWH and warfarin showed that a shorter duration from PVT diagnosis to anticoagulation treatment is associated with better treatment response [20]. In this study, which focused on dalteparin therapy, although duration was not a significant factor in the initial treatment response, it was the only factor associated with the risk of relapse. When comparing the period (three months vs. six months), the more extended period showed a higher risk of relapse, even though it was not proportional to the period. Therefore, this result indicated that early initiation of dalteparin treatment after diagnosis of PVT is an essential factor influencing not only the initial treatment response but also the risk of relapse. 

Although the use of LMWH has been recommended for the treatment of PVT in LC patients, few studies on the proper regimen of LMWH have been conducted. Only previous research on enoxaparin dose for the treatment of PVT in cirrhosis patients directly compared two different dosing regimens that the Food and Drug Administration approved for the therapy of deep venous thrombosis; 1.5 mg/kg subcutaneously (SC) every 24 h and 1 mg/kg SC every 12 h [21]. There was no difference in the therapeutic effect between these two therapies, but 1 mg/kg SC every 12 h, which showed a lower rate of non-variceal bleeding, was evaluated as a better option in anticoagulation therapy.

In this study, we evaluated the efficacy and safety of two different dalteparin dosing regimens, the kg-based regimen and the fixed dosing regimen, for PVT treatment, retrospectively. Between the two groups, the kg-based regimen showed a better treatment response than the fixed dosing regimen for PVT treatment after six months of treatment duration. As there was no difference between the two dosing regimens in bleeding tendency, the kg-based regimen was evaluated as the better choice for anticoagulant therapy for the treatment of PVT in LC patients. 

Bleeding is a significant complication of anticoagulant therapy [20,21,22]. All anticoagulants, including VKAs, LMWH, and DOAC, usually have high major bleeding rates of 1–3% [23]. Bleeding is also a major concern in anticoagulation for the treatment of LC patients with PVT. Considering the characteristics of LC patients who easily shift between bleeding and coagulation, bleeding may occur more frequently when anticoagulation is administered. The previous study reported that 5 out of 55 patients with LC who received anticoagulation therapy for PVT treatment showed bleeding complications related to anticoagulation therapy [20]. In our study, bleeding complications occurred in 11.4% of patients using dalteparin, which was a similar rate to the previous investigation. 

Suppurative thrombosis of the portal vein, which is also called pylephlebitis, is a rare complication of intra-abdominal infection [24]. Treatment of pylephlebitis is based on the administration of broad-spectrum antibiotics to the infection [25,26]. In the case of anticoagulation, its role in treating pylephlebitis has not been established, unlike PVT. Previous research in pylephlebitis patients has reported that the use of anticoagulation increased PVT resolution, resulting in long-term benefits through the prevention of chronic portal hypertension [24]. Based on the favorable outcome, the anticoagulation treatment rate for pylephlebitis has increased from 35–70% to 76.7–82.0% in recent years [27]. In our study, patients with pylephlebitis were not included. Considering the clinical condition of patients with pylephlebitis, which is different from that of PVT patients, caution is needed in applying our findings to them. 

The main limitation of our study is the retrospective design. Since the dosing regimen of dalteparin could not be randomized among patients, selection bias may have affected the study. The lack of data likely occurred because the data were obtained from medical records retrospectively. However, this is the first study to investigate the proper dose of dalteparin therapy for PVT and the predictive factors for relapse in patients after PVT treatment. Further validation with additional prospective studies is needed because of the retrospective study design. 

## 5. Conclusions

Our study demonstrated that the history of variceal bleeding, the status of the thrombus, and the duration from diagnosis of PVT to dalteparin treatment were predictive factors for dalteparin treatment response and relapse. In the dosing regimen, the kg-based regimen, which was the same as the VTE regimen, was a better option for the efficacy and safety of dalteparin therapy. Additionally, when treating PVT in LC patients, careful monitoring is recommended for patients with predictive factors for treatment response and relapse of PVT. 

## Figures and Tables

**Table 1:** Predictive factors for response to dalteparin treatment: univariate analysis.

		No Response (*n* = 41)		Response (*n* = 80)		*p*
		N (or Mean)	% (or ±S.D)	N (or Mean)	% (or ±S.D)	
Sex	Female	11	39.3	17	60.7	0.491
	Male	30	32.3	63	67.7	
Age (year)	<65	25	41.7	35	58.3	0.073
	≥65	16	26.2	45	73.8	
Height (cm)	<165	19	34.5	36	65.5	0.984
	≥165	22	34.4	42	65.6	
Body weight (kg)	<65	25	38.5	40	61.5	0.281
	≥65	16	29.1	39	70.9	
Platelet count (×10^3^/mm^3^) ^a^		73.70	47.23	89.26	54.18	0.122
Prothrombin time (INR) ^a^		1.26	0.17	1.22	0.15	0.269
Cause of LC	Alcoholic	4	36.3	7	63.6	0.725
	Hepatitis B or C	31	32.3	65	37.7	
	Others	6	42.9	8	57.1	
Combined HCC	No	11	35.5	20	64.5	0.827
	Yes	30	33.3	60	66.7	
TACE or RFA	No	13	37.1	22	62.9	0.629
	Yes	28	32.6	58	67.4	
History of variceal bleeding	No	11	17.5	52	82.5	<0.001
	Yes	30	51.7	28	48.3	
Esophageal/gastric varices	No	6	25.0	18	75.0	0.304
	Yes	35	36.1	62	63.9	
Extensive of thrombus	Partial	36	31.9	77	68.1	0.077
	Complete	5	62.5	3	37.5	
Status of thrombus	Newly developed	11	20.4	43	79.6	0.019
	Stationary	1	50.0	1	50.0	
	Progressive	29	44.6	36	55.4	
Child-Pugh classification	A	17	26.6	47	73.4	0.071
	B or C	24	42.1	33	57.9	
Duration from diagnosis of PVT to dalteparin treatment (month)	<6	20	25.6	58	74.4	0.010
	≥6	21	48.8	22	51.1	
Dosing regimen	Fixed	21	45.7	25	54.3	0.032
	kg based	20	26.7	55	73.3	

^a^ A platelet count and a prothrombin time were measured before the initiation of dalteparin. HCC, hepatocellular carcinoma; INR, international normalized ratio; LC, liver cirrhosis; PVT, portal vein thrombosis; RFA, radio-frequency ablation; TACE, trans-catheter arterial chemoembolization.

**Table 2:** Predictive factors for response to dalteparin treatment: multivariate analysis.

Characteristics		Unadjusted OR (95% CI)	Adjusted OR (95% CI)
Male		2.134 (0.663–6.870)	
Age (year)	≥65	2.250 (0.837–6.050)	
Child–Pugh classification	A	2.141 (0.854–5.371)	2.232 (0.920–5.412)
History of variceal bleeding	No	4.278 (1.706–10.728) **	4.633 (1.879–11.426) **
Extensive of thrombus	Partial	4.070 (0.687–24.126)	4.982 (0.927–26.783)
Status of thrombus	Newly developed	2.958 (0.961–9.105)	3.159 (1.236–8.076) *
	Stationary	8.09 (0.250–261.697)	4.297 (0.206–89.803)
	Progressive	0	
Duration from diagnosis of PVT to dalteparin treatment (month)	<6	1.183 (0.388–3.604)	
Dosing regimen	Kg-based	2.310 (1.066–5.007) *	2.597 (1.057–6.383) *

* *p* < 0.05, ** *p* < 0.01 CI, confidence interval; PVT, portal vein thrombosis; OR, odds ratio.

**Table 3:** Predictive factors for relapse of Portal vein thrombosis after dalteparin treatment: univariate analysis.

		No Relapse (*n* = 48)		Relapse (*n* = 32)		*p*
		N (or Mean)	% (or ±S.D)	N (or Mean)	% (or ±S.D)	
Sex	Female	9	52.9	8	47.1	0.503
	Male	39	61.9	24	38.1	
Age (year)	<65	24	68.6	11	31.4	0.168
	≥65	24	53.3	21	46.7	
Platelet count (×10^3^/mm^3^) ^a^		96.44	53.36	78.50	54.47	0.148
Prothrombin time (INR) ^a^		1.20	0.14	1.26	0.15	0.103
Combined HCC	No	9	45	11	55	0.114
	Yes	39	65	21	35	
TACE or RFA	No	11	50	11	50	0.261
	Yes	37	63.8	21	36.2	
History of variceal bleeding	No	33	63.5	19	36.5	0.389
	Yes	15	53.6	13	46.4	
Esophageal/gastric varices	No	12	55.7	6	33.3	0.512
	Yes	36	58.1	26	41.9	
Thrombus filling	Partial	47	61	30	39	0.337
	Complete	1	33.3	2	66.7	
Status of thrombus	Newly developed	26	60.5	17	39.5	0.700
	Stationary	1	100	0	0	
	Progressive	21	58.3	15	41.7	
Child–Pugh classification	A	26	55.3	21	44.7	0.308
	B or C	22	66.7	11	33.3	
Duration from diagnosis of PVT to dalteparin treatment (month)	<3	35	70.0	15	30.0	0.018
	≥3	13	43.3	17	56.7	
Duration from diagnosis of PVT to dalteparin treatment (month)	<6	40	69.0	18	31.0	0.008
	≥6	8	36.4	14	63.6	
Dosing regimen	Fixed	18	72.0	7	28.0	0.140
	Kg-based	30	54.5	25	45.5	
Duration of therapy (month)	<6	15	78.9	4	21.1	0.645
	≥6	33	54.1	28	45.9	

^a^ A platelet count and a prothrombin time were measured before the initiation of dalteparin. HCC, hepatocellular carcinoma; INR, international normalized ratio; PVT, portal vein thrombosis; RFA, radio-frequency ablation; TACE, trans-catheter arterial chemoembolization.

**Table 4:** Predictive factors for relapse of Portal vein thrombosis after dalteparin treatment: multivariate analysis.

Characteristics		Unadjusted OR (95% CI)	Model I	Model II
			Adjusted OR (95% CI)	Adjusted OR (95% CI)
Male		0.692 (0.235–2.038)		
Age (year)	≥65	1.909 (0.758–4.806)		
Duration from diagnosis of PVT to dalteparin treatment (month)	≥3	3.051 (1.190–7.827) *	3.051 (1.190–7.827) *	
Duration from diagnosis of PVT to dalteparin treatment (month)	≥6	3.889 (1.386–10.910) *		3.889 (1.386–10.910) *

* *p* < 0.05 For model I construction, sex, age, and duration from diagnosis of PVT to dalteparin treatment within three months were included for analysis. For model II construction, sex, age, and duration from diagnosis of PVT to dalteparin treatment within six months were included. Cl, confidence interval; PVT, portal vein thrombosis; OR, odds ratio.

**Table 5:** Clinical characteristics in two dosing regimens during dalteparin therapy.

Characteristics			Fixed Dosing Regimen (*n* = 46)		Kg-Based Dosing Regimen (*n* = 75)		*p*
			N	%	N	%	
Treatment response	Yes	Complete	10	33.3	20	33.7	0.032
		Partial	15	30.0	35	70.0	
	No	Stable or progressive	21	51.2	20	48.8	
Bleeding	Yes		3	21.4	11	78.6	0.174
	No		43	40.2	64	59.8	
Sex	Female		10	35.7	18	64.3	0.775
	Male		36	38.7	57	61.3	
Age (year)	<65		28	46.7	32	53.3	0.052
	≥65		18	29.5	43	70.5	
Child-Pugh classification	A		25	39.1	39	60.9	0.802
	B or C		21	36.8	36	63.2	
Combined HCC	No		13	41.9	18	58.1	0.602
	Yes		33	36.7	57	63.3	
TACE or RFA	No		14	40.0	21	60.0	0.774
	Yes		32	37.2	54	62.8	
History of variceal bleeding	No		24	38.1	39	61.9	0.985
	Yes		22	37.9	36	62.1	
Esophageal/gastric varices	No		11	45.8	13	54.2	0.378
	Yes		35	36.1	62	63.9	
Thrombus filling	Partial		42	37.2	71	62.8	0.470
	Complete		4	50.0	4	50.0	
Status of thrombus	Newly developed		19	35.2	35	64.8	0.813
	Stationary		1	50.0	1	50.0	
	Progressive		26	40.0	39	60.0	

HCC, hepatocellular carcinoma; PVT, portal vein thrombosis; RFA, radio-frequency ablation; TACE, trans-catheter arterial chemoembolization.

## Data Availability

The data that support the findings of this study are available on request from the corresponding author. The data are not publicly available due to their containing information that could compromise the privacy of research participants.
